# Ferroptosis-related gene HIC1 in the prediction of the prognosis and immunotherapeutic efficacy with immunological activity

**DOI:** 10.3389/fimmu.2023.1182030

**Published:** 2023-06-14

**Authors:** Yanlin Wu, Zhengjun Lin, Xianzhe Tang, Zhongyi Tong, Yuqiao Ji, Yingting Xu, Ziting Zhou, Jing Yang, Zhihong Li, Tang Liu

**Affiliations:** ^1^ Department of Orthopedics, The Second Xiangya Hospital, Central South University, Changsha, Hunan, China; ^2^ Department of Orthopedics, Chenzhou No.1 People’s Hospital, Chenzhou, Hunan, China; ^3^ Department of Pathology, The Second Xiangya Hospital, Central South University, Changsha, Hunan, China

**Keywords:** HIC1, pan-cancer, genetic alternation, prognosis, immune microenvironment, immunotherapeutic efficacy, drug sensitivity

## Abstract

**Background:**

Hypermethylated in Cancer 1 (HIC1) was originally confirmed as a tumor suppressor and has been found to be hypermethylated in human cancers. Although growing evidence has supported the critical roles of HIC1 in cancer initiation and development, its roles in tumor immune microenvironment and immunotherapy are still unclear, and no comprehensive pan-cancer analysis of HIC1 has been conducted.

**Methods:**

HIC1 expression in pan-cancer, and differential HIC1 expression between tumor and normal samples were investigated. Immunohistochemistry (IHC) was employed to validate HIC1 expression in different cancers by our clinical cohorts, including lung cancer, sarcoma (SARC), breast cancer, and kidney renal clear cell carcinoma (KIRC). The prognostic value of HIC1 was illustrated by Kaplan-Meier curves and univariate Cox analysis, followed by the genetic alteration analysis of HIC1 in pan-cancer. Gene Set Enrichment Analysis (GSEA) was conducted to illustrate the signaling pathways and biological functions of HIC1. The correlations between HIC1 and tumor mutation burden (TMB), microsatellite instability (MSI), and the immunotherapy efficacy of PD-1/PD-L1 inhibitors were analyzed by Spearman correlation analysis. Drug sensitivity analysis of HIC1 was performed by extracting data from the CellMiner™ database.

**Results:**

HIC1 expression was abnormally expressed in most cancers, and remarkable associations between HIC1 expression and prognostic outcomes of patients in pan-cancer were detected. HIC1 was significantly correlated with T cells, macrophages, and mast cell infiltration in different cancers. Moreover, GSEA revealed that HIC1 was significantly involved in immune-related biological functions and signaling pathways. There was a close relationship of HIC1 with TMB and MSI in different cancers. Furthermore, the most exciting finding was that HIC1 expression was significantly correlated with the response to PD-1/PD-L1 inhibitors in cancer treatment. We also found that HIC1 was significantly correlated with the sensitivity of several anti-cancer drugs, such as axitinib, batracylin, and nelarabine. Finally, our clinical cohorts further validated the expression pattern of HIC1 in cancers.

**Conclusions:**

Our investigation provided an integrative understanding of the clinicopathological significance and functional roles of HIC1 in pan-cancer. Our findings suggested that HIC1 can function as a potential biomarker for predicting the prognosis, immunotherapy efficacy, and drug sensitivity with immunological activity in cancers.

## Introduction

Cancer is a great threat to human health and is one of the major causes of death, which ubiquitously affects people globally and brings a great economic burden to society (1). Immunotherapy, mainly including immune checkpoint inhibitors (ICIs) and adoptive cell therapy (ACT), has led to the revolution of anti-cancer treatments and attracted the attention of tumor immunology (2). However, only a fraction of cancer patients can respond to current cancer immunotherapies, and most patients have innate or acquired immunotherapeutic resistance (3, 4). The tumor immune microenvironment, including tumor-infiltrating immune cells, and immune-related biomolecules, is critically involved in cancer initiation and development, and recent work has verified novel targets in the tumor immune microenvironment for cancer immunotherapy (5). By dissecting the mechanisms underlying cancer immunotherapy resistance, the tumor immune microenvironment has been confirmed as a major location for immunoresistance to occur (6). Therefore, it is warranted to explore critical modulators mediating the tumor immune microenvironment and novel biomolecules to predict the immunotherapeutic efficacy of cancer patients.

Hypermethylated in Cancer 1 (HIC1), located on chromosome 17p13.3 completely within a CpG island, is a tumor repressor that is widely expressed in normal tissues, however, is generally lowly expressed with methylation in several cancers, such as prostate cancer, breast cancer, and pancreatic cancer (7–9). In 1995, HIC1 was first discovered and was found to be activated by p53 (10). Chen et al. indicated that the loss of HIC1 function could induce the development of cancer by activating the deacetylase SIRT1, subsequently downregulating the expressions of p53 (11). Interacting with several major repression and chromatin remodeling complexes, including CtBP, NuRD, PRC2, and SWI/SNF, HIC1 is recognized as a multifaceted transcriptional repressor. Besides, it has been found that HIC1 is involved in multiple physiological processes and oncology, such as embryonic development, DNA damage repair, and angiogenesis (12). For instance, the abundant methylation status of 11 CpG sites within the HIC1 promoter has been detected in cell lines, tissues, and plasma of patients with prostate cancer compared with normal controls. Restoration of HIC1 expression could suppress the proliferation, migration, and invasion and induce the apoptosis of prostate cancer cells (7). In bladder cancer, ZBTB7A can bind to the HIC1 promoter, and decreased HIC1 expression can promote the malignant behavior of bladder cancer cells (13). Recent work has suggested the regulatory roles of HIC1 in ferroptosis during cancer progression. It has been found that HIC1 controlled several pro-ferroptosis genes transcriptionally, such as HBA1, and promotes ferroptosis in liver cancer (14). Notably, several studies have reported controversial findings indicating the potential oncogenic functions of HIC1 (15). Generally, HIC1 plays a critical role in various cancers, however, there is no pan-cancer analysis of HIC1 and the immune-mediating functions of HIC1 in cancers are largely unknown.

In this research, we presented and validated the HIC1 expression landscape in different cancers, and its association with the prognosis of cancer patients was also explored. Moreover, we also explored the genetic alternation characteristics and the potential biological functions and signaling pathways of HIC1. Furthermore, the potential functions of HIC1 in mediating the tumor immune microenvironment and predicting the immunotherapeutic efficacy and drug sensitivity were further investigated. Our results highlighted that HIC1 plays an important role in the progression and therapy of various cancers, thereby offering new insight into cancer immunotherapy.

## Materials and methods

### Data collection

The normalized TCGA pan-cancer dataset was downloaded from the UCSC database (https://xena.ucsc.edu/) (16), and the expression data of HIC1 of each sample in 33 cancers was extracted. In addition, expression profiles of different cancer cell lines were also downloaded from the Broad Institute Cancer Cell Line Encyclopedia (CCLE) portal database (https://portals.broadinstitute.org/ccle/about) and HIC1 expression levels in 21 cancer cell lines were also investigated. Moreover, the expression levels of HIC1 in normal tissues were assessed by expression profiles from Genotype-Tissue Expression (GTEx) database (https://www.gtexportal.org/). Differential expression analysis between cancer samples and their corresponding normal samples in the TCGA pan-cancer. Besides, we also confirmed the differential expression of HIC1 between tumor samples in the TCGA pan-cancer database and normal samples in the GTEx database. Finally, we explored differential HIC1 expression among patients with different clinical stages. The abbreviations of 33 cancer types are presented in **Table S1**.

### Prognostic analysis of HIC1

The correlation of HIC1 expression with overall survival (OS), disease-specific survival (DSS), disease-free survival (DFS), and progression-free survival (PFS) was evaluated through utilizing TCGA pan-cancer survival data by univariate Cox regression analysis, and the results were visualized by forest map. Kaplan–Meier curves with log-rank p values were further employed to illustrate the differential survival outcomes between of high-HIC1 expression and low-HIC1 expression groups in different cancers. R-packages “survival”, “survminer”, “forestplot”, “limma” and “ggpubr” were utilized for this investigation process.

### Genetic alternation analysis of HIC1

The genetic mutation characteristics of HIC1 were investigated by utilizing”TCGA Pan-Cancer Atlas Studies” dataset in the online database cbioportal (cBio Cancer Genomics Portal) (http://cbioportal.org) (17). The genetic alteration frequency, mutation type, and copy number alteration (CNA) of HIC1, the mutated sites, and the three-dimensional structure of HIC1 were investigated.

### Immune microenvironment assessment

Estimation of Stromal and Immune cells in Malignant Tumor tissues using Expression data (ESTIMATE) analysis was employed to calculate the stromal and immune scores of each tumor sample by “estimate” R package (18). CIBERSORT, a bioinformatics algorithm that can quantify the immune cellular composition of tissue samples according to their gene expression levels, was utilized to explore the correlation between HIC1 and diverse immune cells within the tumor immune microenvironment in different cancer types (18). The relationship between HIC1 expression and infiltrating immune cells was evaluated by utilizing Spearman correlation analysis. TISIDB (http://cis.hku.hk/TISIDB/index.php) is an integrated online portal for the investigation of tumor-immune system interaction. We utilized TISIDB online database to determine the relationship between HIC1 expression and tumor-infiltrating lymphocyte (TILs) expression, major histocompatibility complex (MHC) genes expression, immunoinhibitory/immunostimulator genes expression, chemokines and chemokines receptors expression in human cancers. |R| >0.5, P-value <0.05 was considered as significantly relative.

### Gene set enrichment analysis

GSEA was conducted to explore the possible biological functions and potential signaling pathways modulated by HIC1 in each cancer type. The “gmt” data of the hallmark gene set (h.all.v7.4.symbols.gmt) which consists of 50 hallmark gene sets was extracted from the MSigDB database (https://www.gsea-msigdb.org/gsea/index.jsp). The analysis process was performed and visualized by utilizing R packages “clusterProfiler” (19), “enrichplot”, and “ggplot2”.

### Investigation of HIC1 in predicting immunotherapeutic efficacy

To assess the connection between HIC1 expression and the immunotherapeutic responses to immune checkpoint blockade (ICB), three datasets providing patients with immunotherapy treatment, including GSE78220 (melanoma) (20), GSE67501 (renal cell carcinoma) (21), and IMvigor210 (metastatic urological cancer) extracted from GEO (https://www.ncbi.nlm.nih.gov/geo/) online database were included in our study. The procedure was conducted and the results were visualized utilizing the R-package “ggpubr” and “ggplot2”.

### Investigation of HIC1 in predicting drug sensitivity

To investigate the correlation between HIC1 expression and drug sensitivity, NCI-60 compound activity data with RNA-seq expression profiles were downloaded from the CellMiner™ online database (https://discover.nci.nih.gov/cellminer/home.do). Drugs approved by FDA were included in our analysis by utilizing R packages “impute”, “limma”, “ggplot2”, and “ggpubr”.

### Clinical samples and immunohistochemistry

Patient samples were obtained under a Second Xiangya Hospital-approved protocol. Informed consent was obtained from all patients in accordance with the Declaration of Helsinki. HIC1 immunostains in all cancer cases were reviewed and evaluated by pathologists ZY T and P Z. Clinical tumor tissue samples and commercially available tumor tissue chips were stained for HIC1. IHC staining was implemented with HIC1 antibody (1:50; Proteintech, China) based on the manufacturer’s protocols. Sections of tumor tissues were deparaffinized and rehydrated. Then, the antigen was retrieved by being immersed in pH=6.0 citrate buffer for 15 minutes at 95°C before incubation with 0.3% hydrogen peroxide for 15 mins at room temperature to block the activity of endogenous peroxidase. Sections were treated with PBS rinsing and 5% normal goat serum blocking for 30 minutes at room temperature before being treated with a primary anti-HIC1 antibody and incubated overnight at 4°C. The proportion of negative (–), weakly positive (+), moderately positive (++), or strongly positive (+++) staining cells and cell staining intensity in five randomly selected fields were counted. The immunoreactivity scores were calculated by multiplying a number representing the percentage of immunoreactive cells (0+, none; 1+, <25%; 2+, 25%-50%; 3+, 51%-75%; and 4+, 75%-100%.) by the number representing staining intensity (0, negative; 1, weak; 2, moderate; 3, strong). The immunoreactivity scores were obtained by multiplying the scores for distribution and intensity, giving scores in the range of 0-12. IHC images of HIC1 protein expression in four tumor tissues, including colon adenocarcinoma (COAD), breast invasive carcinoma (BRCA), lung squamous cell carcinoma (LUSC), lung adenocarcinoma (LUAD), and their corresponding normal tissues were also downloaded from the HPA database (http://www.proteinatlas.org/). The IHC results were also compared with the protein level of HIC1 in TCGA from the UALCAN database (https://ualcan.path.uab.edu/).

### Statistical analysis

All statistical analyses were conducted in R programming, version 4.1.1. The Wilcoxon rank-sum test was used to calculate the gene expression and the methylation level differences between cancerous and normal tissues of each cancer type. The coefficient values were evaluated by Spearman correlation analysis. P < 0.05 was considered statistically significant (*p < 0.05, **p < 0.01, and ***p < 0.001).

## Results

### The expression pattern of HIC1 in pan-cancer

**Figure 1:**
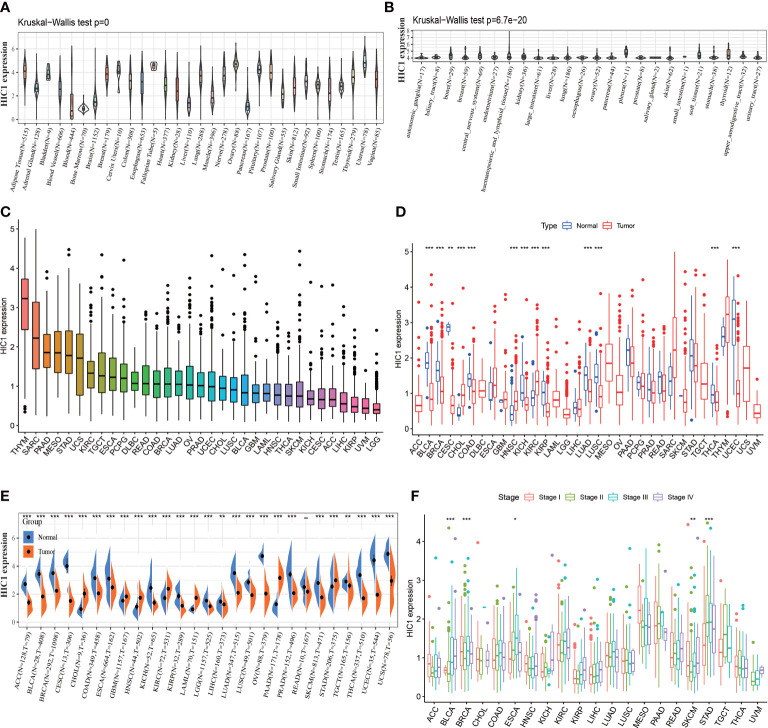
The expression pattern of HIC1. **(A)** The expression level of HIC1 in 31 normal tissues from the GTEx database. **(B)** The expression level of HIC1 in 24 tumor cell lines from the CCLE database. **(C)** The expression level of HIC1 in pan-cancer. **(D)** Comparison of HIC1 expression level between cancer and normal samples from TCGA database. **(E)** Comparison of HIC1 expression level between cancer and normal samples from GTEx database. **(F)** The expression level of HIC1 in patients with different WHO stages in various cancer from the TCGA database. *p < 0.05, **p < 0.01, ***p < 0.001.

**Figure 2:**
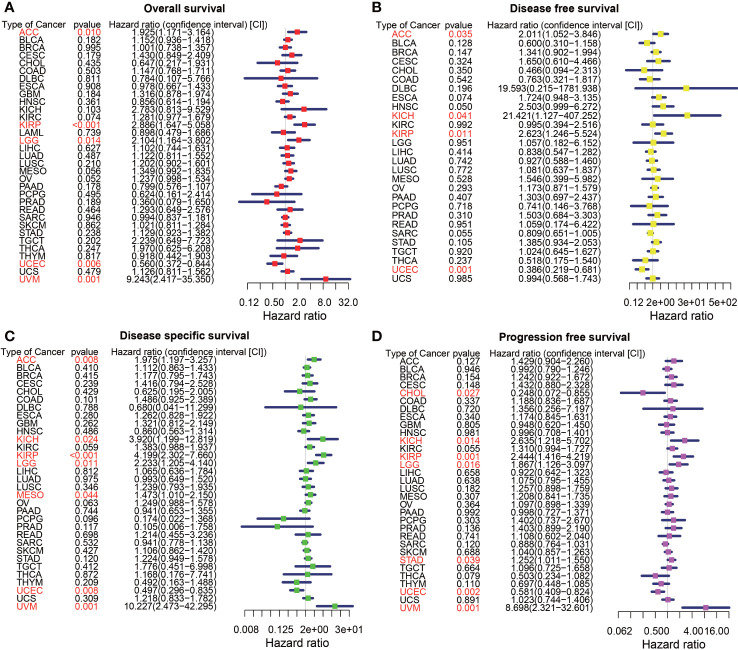
The forest map of univariate Cox regression analysis of HIC1. **(A)** The forest map shows the results of univariate Cox regression analysis of HIC1 for OS in TCGA pan-cancer. **(B)** The forest map shows the results of univariate cox regression analysis of HIC1 for DFS in TCGA pan-cancer. **(C)** Forest map shows the results of univariate Cox regression analysis of HIC1 for DSS in TCGA pan-cancer. **(D)** The forest map shows the results of univariate cox regression analysis of HIC1 for PFS in TCGA pan-cancer. Red items indicate statistical significance.

To explore the expression levels of HIC1 across normal tissues and cancers, we analyzed the HIC1 expression of samples in GTEx, CCLE, and TCGA pan-cancer databases. The investigation of HIC1 expression in the GTEx database found that HIC1 was highly expressed in several tissues, such as ovary, uterus, and breast tissues, while was lowly expressed in bone marrow, liver, and pancreas tissues in comparison with other normal tissue samples (**Figure 1A**). The expression of HIC1 in different cancer cell lines was shown in **Figure 1B**, which showed that HIC1 was highly expressed in bone, central nervous system, and pleura cancer cell lines compared with other cancer cell lines. As for HIC1 expression in the TCGA pan-cancer dataset, the results showed that HIC1 was highly expressed in thymoma (THYM) and SARC, while was lowly expressed in brain lower grade glioma (LGG) and uveal melanoma (UVM) compare to other cancer types (**Figure 1C**). Differential expression analysis indicated that HIC1 expression was strongly decreased in tumor samples in comparison with their compared normal samples of TCGA pan-cancer dataset in bladder urothelial carcinoma (BLCA), BRCA, cervical squamous cell carcinoma, and endocervical adenocarcinoma (CESC), COAD, kidney chromophobe (KICH), kidney renal papillary cell carcinoma (KIRP), LUAD, LUSC, thyroid carcinoma (THCA) and uterine corpus endometrial carcinoma (UCEC), while was significantly increased in cholangiocarcinoma (CHOL), head and neck squamous cell carcinoma (HNSC), and KIRC (**Figure 2D**). Moreover, we also compared the differential expression between cancer samples and their corresponding normal samples in the GTEx database. The results showed that HIC1 was abnormally higher in cancer samples in CHOL, glioblastoma multiforme (GBM), HNSC, KIRC, acute myeloid leukemia (LAML), pancreatic adenocarcinoma (PAAD), and stomach adenocarcinoma (STAD), while was significantly downregulated in adrenocortical carcinoma (ACC), BLCA, BRCA, CESC, COAD, esophageal carcinoma (ESCA), KICH, LGG, LICH, LUAD, LUSC, ovarian serous cystadenocarcinoma (OV), prostate adenocarcinoma (PRAD), skin cutaneous melanoma (SKCM), testicular germ cell tumors (TGCT), THCA, UCEC, and uterine carcinosarcoma (UCS) (**Figure 1E**). These results indicated that HIC1 expression is abnormally high or low in various types of cancer, suggesting that HIC1 may play a potentially important role in cancer diagnosis. Furthermore, we also investigated the associations of HIC1 with clinical stages in patients with different cancers, and the results indicated patients in advanced clinical stages presented higher HIC1 expression levels in BLCA, ESCA, and STAD, and significant differences in the HIC1 expression among patients with different clinical stages were also detected in BRCA and SKCM (**Figure 1F**).

### Prognostic significance of HIC1

To explore the prognostic significance of HIC1 in pan-cancer, we first conducted the univariate Cox regression analysis to illustrate the associations of HIC1 with OS, DFS, DSS, and PFS in different cancer types. The forest map showed that HIC1 expression was correlated with OS in ACC, KIRP, LGG, UCEC, and UVM. HIC1 was associated with worse OS in ACC (HR, 1.925; 95% CI, 1.171-3.164; P = 0.010), KIRP (HR, 2.886; 95% CI, 1.647-5.058; P < 0.001), LGG (HR, 2.104; 95% CI, 1.164-3.802; P =0.014), and UVM (HR, 9.243; 95% CI, 2.417-35.350; P = 0.001), while was correlated with better OS in UCEC (HR, 0.560; 95% CI, 0.372-0.844; P = 0.006) in the assessment of OS in pan-cancer (**Figure 2A**). For univariate Cox analysis of DFS, the results indicated that HIC1 was a risk factor in ACC (HR, 2.011; 95% CI, 1.052-3.846; P = 0.035), KICH (HR, 21.421; 95% CI, 1.127-407.252; P = 0.041), and KIRP (HR, 2.623; 95% CI, 1.246-5.524; P = 0.011), while was a protective factor in UCEC (HR, 0.386; 95% CI, 0.219-0.681; P = 0.001) (**Figure 2B**). The forest map of DSS showed that HIC1 expression was correlated with worse DSS in ACC (HR, 1.975; 95% CI, 1.197-3.257; P = 0.008), KICH (HR, 3.920; 95% CI, 1.199-12.819; P = 0.024), KIRP (HR, 4.199; 95% CI, 2.302-7.660; P < 0.001), LGG (HR, 2.233; 95% CI, 1.205-4.140; P = 0.011), mesothelioma (MESO) (HR, 1.473; 95% CI, 1.010-2.150; P = 0.044) and UVM (HR, 10.227; 95% CI, 2.473-42.295; P = 0.001), while was associated with better DSS in UCEC (HR, 0.497; 95% CI, 0.296-0.835; P = 0.008) (**Figure 2C**). With regards to PFS, there was a close relationship between HIC1 expression and PFS in CHOL, KICH, KIRP, LGG, STAD, UCEC, and UVM, and HIC1 could serve as a risk regulator for PFS in KICH (HR, 2.635; 95% CI, 1.218-5.702; P = 0.014), KIRP (HR, 2.444; 95% CI, 1.416-4.219; P = 0.001), LGG (HR, 1.867; 95% CI, 1.126-3.097; P = 0.016), STAD (HR, 1.252; 95% CI, 1.011-1.550; P = 0.039), and UVM (HR, 8.698; 95% CI, 2.321-32.601; P = 0.001), while could serve as a protective regulator for PFS in CHOL (HR, 0.248; 95% CI, 0.072-0.855; P = 0.027) and UCEC (HR, 0.581; 95% CI, 0.409-0.824; P = 0.002) (**Figure 2D**).

**Figure 3:**
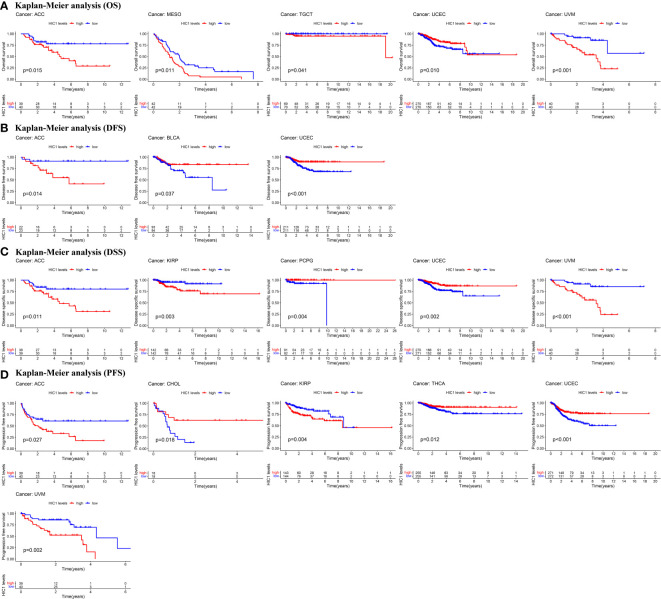
Kaplan-Meier survival curves of HIC1 in pan-cancer. **(A)** Kaplan–Meier analysis of the correlation between HIC1 expression and OS in 5 cancer types. **(B)** Kaplan–Meier analysis of the correlation between HIC1 expression and DFS in 3 cancer types. **(C)**Kaplan–Meier analysis of the correlation between HIC1 expression and DSS in 5 cancer types. **(D)** Kaplan–Meier analysis of the correlation between HIC1 expression and PFS in 7 cancer types.

Next, Kaplan–Meier curves were drawn to compare the differences in the survival time between high HIC1 expression and low HIC1 expression subgroups. The OS Kaplan–Meier curves indicated that patients with high HIC1 expression in ACC (P = 0.015), MESO (P = 0.011), TGCT (P = 0.041), and UVM (P < 0.001) had a shorter survival time, while UCEC patients with high HIC1 expression had a longer survival time (P = 0.010) (**Figure 3A**). As for DFS Kaplan–Meier curves, we detected that HIC1 expression was linked to shorter survival time in ACC (P = 0.014), whereas was associated with longer survival time in BLCA (P = 0.037) and UCEC (P < 0.001) (**Figure 3B**). With regards to DSS Kaplan–Meier curves, we found that HIC1 expression was significantly connected with poor DSS in ACC (P = 0.011), KIRP (P = 0.003), and UVM (P < 0.001), while was related to better DSS in pheochromocytoma and paraganglioma (PCPG) (P = 0.004) and UCEC (P = 0.002) (**Figure 3C**). Finally, the Kaplan–Meier curves of PFS showed that high HIC1 expression predicted poor PFS in ACC (P = 0.027), KIRP (P = 0.004), and UVM (P = 0.002), while predicted better PFS in CHOL (P = 0.018), THCA (P = 0.012) and UCEC (P < 0.001) (**Figure 3D**). In summary, these results indicated that HIC1 may function as a prognosis-related risk factor in several cancers, including ACC, MESO, KIRP, TGCT, and UVM, and a prognosis-related protective factor in BLCA, CHOL, PCPG, THCA, and UCEC.

### Genetic alternation analysis of HIC1

**Figure 4:**
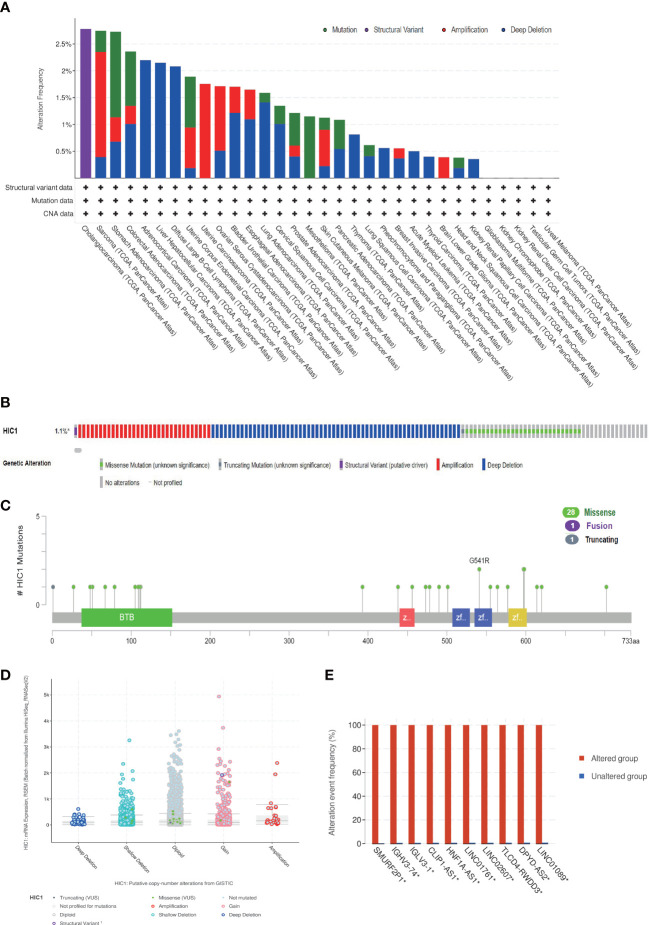
The genetic alteration characteristics of HIC1 in pan-cancer. **(A)** The alteration frequency of HIC1 with different types of mutations in different cancer types. **(B)** Different genetic alteration types of HIC1. **(C)** The mutation types, sites, and sample numbers of the HIC1 genetic alterations. **(D)** The correlated alteration types and putative copy-number of HIC1 in pan-cancer. **(E)** Co-occurrence of genetic mutations in tumors with HIC1 alterations.

Next, we investigated the genetic alternation characteristics of HIC1 in the cbioportal database. The genetic alternation frequency of HIC1 was approximately 1.1%, and the genetic alternation frequency was higher than 2.5% in 3 cancer types, including CHOL, SARC, and STAD in TCGA pan-cancer cohort (**Figure 4A**). Deep deletion, amplification, and missense mutation were the major types of genetic alteration of HIC1 in pan-cancer (**Figure 4B**). Furthermore, we investigated the genetic mutation types, sites, and case samples of HIC1. Missense mutation was the most common alternation type of HIC1, while G541R mutation was detected in two samples in PRAD and STAD respectively (**Figure 4C**). In addition, the putative copy-number alterations of HIC1 from genomic identification of significant targets in cancer (GISTIC) included many types, such as deep deletion, shallow deletion, amplification, and gain function, contributing to the alternations of gene expression (**Figure 4D**). The genetic alterations of SMURF2P1, IGHV3-74, IGLV3-1, CLIP1-AS1, HNF1A-AS1, LINC01761, LINC02607, TLCD4-RWDD3, DPYD-AS2, and LINC01089 were more commonly occurred in the HIC1-altered group in comparison with unaltered group (**Figure 4E**).

### Gene set enrichment analysis

**Figure 5:**
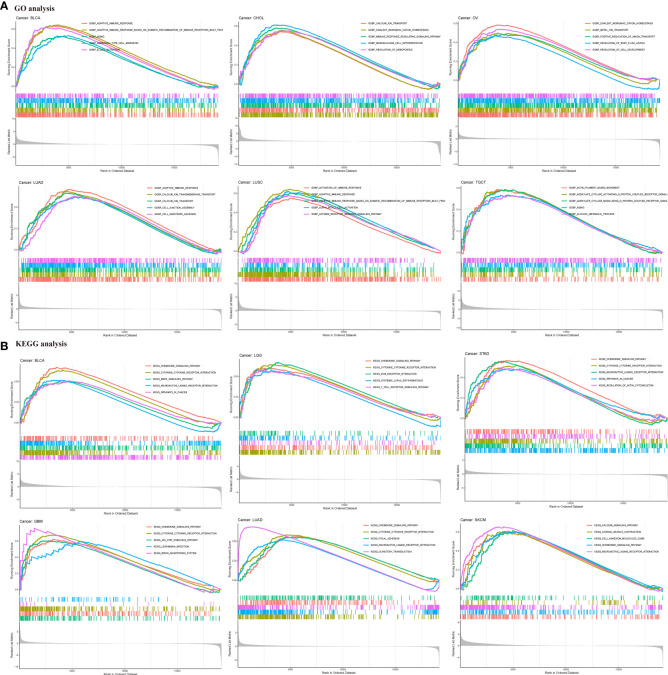
GSEA of HIC1. **(A)** GO functional annotation of HIC1 shows that HIC1 might exert biological functions in calcium ion transport in cancer, and modulating immune system. **(B)** KEGG pathway analysis of HIC1 indicated HIC1 was most commonly involved in the chemokine signaling pathway and cytokine-cytokine receptor interaction, as well as T cell receptor signaling pathway, calcium signaling pathway, JAK-STAT signaling pathway in different cancer types. Peaks on the upward curve indicate positive regulation and peaks on the downward curve indicate negative regulation.

To investigate the potential biological functions and signaling pathways of HIC1 in the specific cancer type, KEGG pathway and GO functional analyses were performed. The results of KEGG analysis indicated that HIC1 was most commonly involved in the chemokine signaling pathway and cytokine-cytokine receptor interaction, as well as the T cell receptor signaling pathway, calcium signaling pathway, JAK-STAT signaling pathway in different cancer types (**Figure 5A**). GO analysis found that HIC1 might exert biological functions in calcium ion transport in cancer, and functions on the immune system, including adaptive immune response, activation of immune response, regulation of lymphocyte activation, and T cell activation in cancer biology (**Figure 5B**). These results indicated that calcium transport and calcium signaling pathway and immune modulatory functions were most commonly involved in HIC1 in cancer biology, suggesting the critical roles of HIC1 in regulating the tumor immune microenvironment.

### Correlation of HIC1 expression with the tumor immune microenvironment

**Figure 6:**
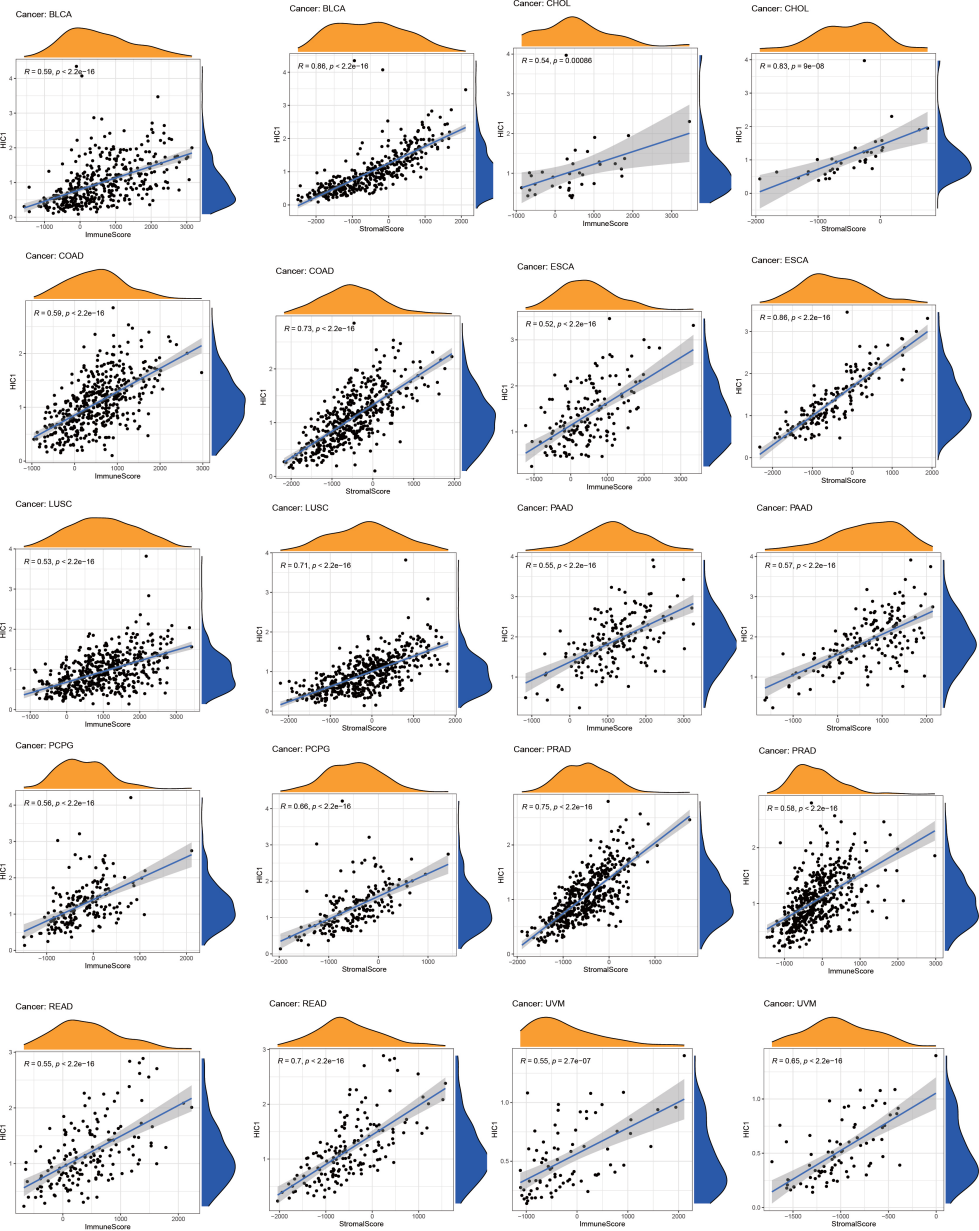
The correlation between HIC1 and immune and stromal scores in pan-cancer.

**Figure 7:**
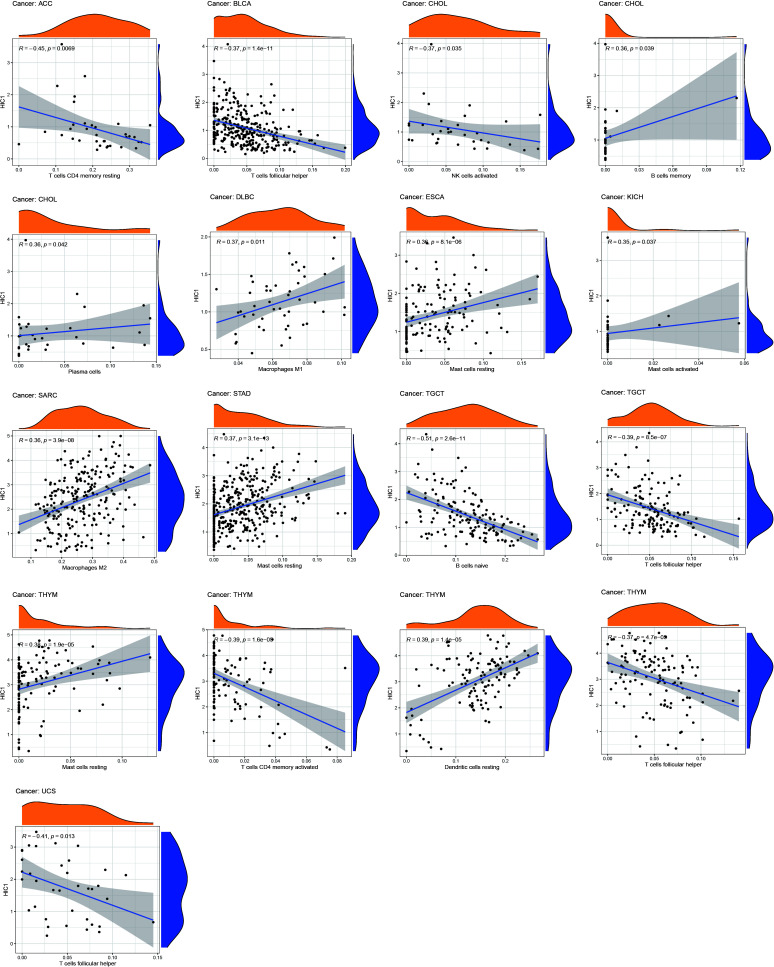
The correlation between HIC1 expression and the immune cells infiltration in pan-cancer.

To further uncover the potential immunomodulatory functions of HIC1 in tumor immunity, we employed the ESTIMATE algorithm, CIBERSORT algorithm, and TISIDB databases to investigate the correlations of HIC1 with the tumor immune microenvironment in pan-cancer. The results of the ESTIMATE algorithm suggested that HIC1 expression was positively correlated with immune and stromal scores in multiple cancers, including BLCA, CHOL, COAD, ESCA, LUSC, PAAD, PCPG, PRAD, rectum adenocarcinoma (READ), and UVM (**Figure 6**). In addition, there was a positive correlation between HIC1 expression and stromal scores in SARC, SKCM, STAD, TGCT, UCEC, OV, liver hepatocellular carcinoma (LIHC), LUAD, KIRP, HNSC, CESC, and BRCA (**Figure S1**). Moreover, we utilized CIBERSORT to analyze the abundance of diverse infiltrating immune cells in the specific cancer type. The results showed that HIC1 expression was negatively related to T follicular helper cells infiltration in UCS, THYM, TGCT, and BLCA, and CD4+ memory resting T cells infiltration in ACC and THYM. In particular, there was also a negative correlation of HIC1 expression with B naive cells abundance in TGCT, NK activated cells abundance in CHOL, whereas a positive correlation of HIC1 expression with mast resting cells in ESCA, STAD, and THYM, mast activated cells in KICH, dendritic resting cells in THYM, M1 macrophages in lymphoid neoplasm diffuse large B-cell lymphoma (DLBC), M2 macrophages in SARC, and plasma cells in CHOL (**Figure 7**). Overall, HIC1 expression was mainly correlated with T cells, B cells, macrophages, and mast cells within the tumor immune microenvironment in multiple cancer types.

**Figure 8:**
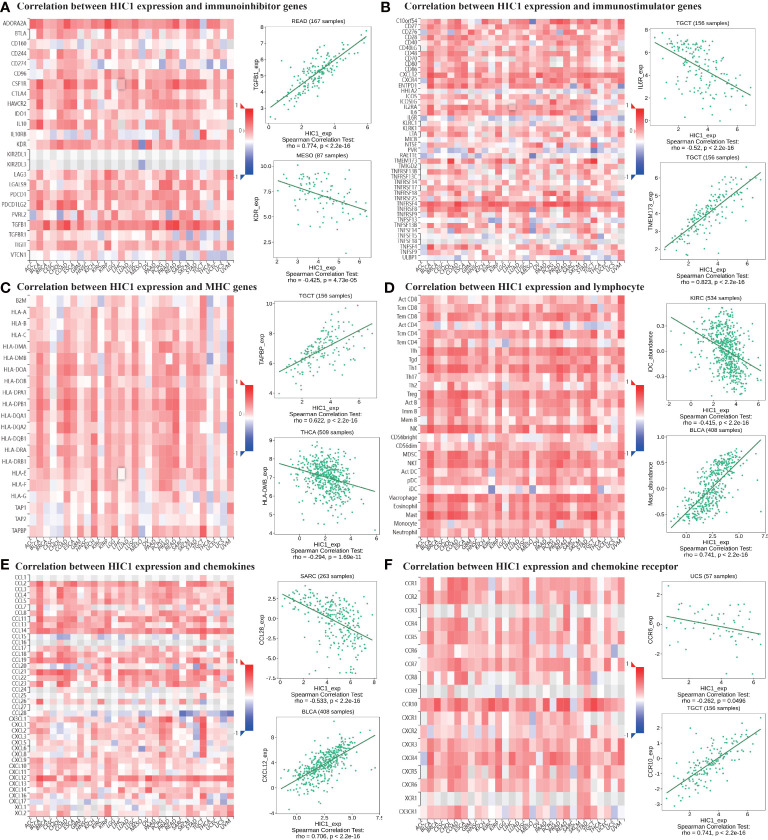
The correlation between HIC1 expression and immune-related biomarkers in the TISIDB database. The co-expression heatmaps show the association between HIC1 expression and **(A)** immunoinhibitor genes, **(B)** immunostimulator genes, **(C)** MHC genes, **(D)** lymphocyte, **(E)** chemokines, and **(F)** chemokines receptors in pan-cancer.

Moreover, TISIDB online database was utilized to explore the effects of HIC1 on mediating tumor-infiltrating lymphocytes, the expression of MHC genes, immunoinhibitory/immunostimulator genes, chemokines, and chemokines receptors during cancer progression. There were positive correlations between HIC1 expression and several immunoinhibitory genes, such as TGFB1, ADORA2A, and CSF1R in multiple cancers (**Figure 8A**). It was also detected that HIC1 expression was positively related to several immunestimulators in different cancers, such as CXCL12 and TNFRSF4, and was negatively connected with IL-6R in TGCT (**Figure 8B**). Besides, we found that HIC1 expression was positively linked to MHC genes, such as TAPBP in TGCT and HLA-DPB1 in COAD (**Figure 8C**). In addition, there were positive correlations between HIC1 and lymphocytes, such as macrophages and mast cells in multiple cancers (**Figure 8D**). With regards to chemokines and chemokines receptors, our results revealed significantly positive correlations of HIC1 with CXCL12 and CCR10 in several cancers (**Figures 8E, F**). These findings revealed that HIC1 may function as an important mediator of immune-related biomolecules and lymphocytes in the tumor immune microenvironment.

### Correlation of HIC1 with TMB and MSI

**Figure 9:**
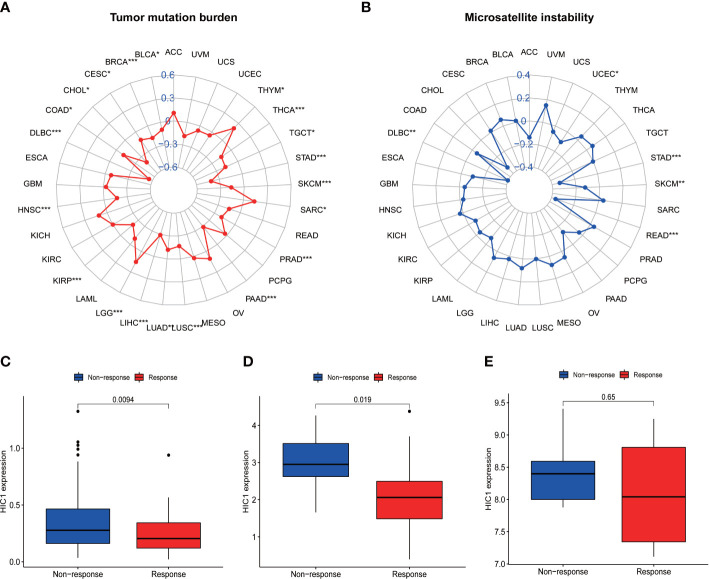
The correlation between HIC1 expression and TMB levels, MSI event, and immunotherapeutic efficacy. **(A)** Radar map of the relationship between HIC1 expression and TMB levels. **(B)** Radar map of the relationship between HIC1 expression and MSI event. **(C–E)** The relationship between HIC1 expression and the immunotherapeutic efficacy in IMvigor210 cohort **(C)**, GSE78220 **(D)**, and GSE67501 **(E)**. *p < 0.05, **p < 0.01; ***p < 0.001.

To illustrate the predictive value of HIC1 in cancer immunotherapy treatment, we further investigated the relationship of HIC1 expression with TMB and MSI, two biomarkers that are closely connected with cancer immunotherapy efficacy. The radar figure showed that HIC1 expression was negatively connected with MSI level in STAD, READ, SKCM, DLBC, and UCEC (**Figure 9B**). As for TMB, there was a significantly negative correlation between HIC1 expression and TMB in multiple cancer types, including THCA, STAD, SKCM, PRAD, PAAD, LUSC, LUAD, LIHC, KIRP, HNSC, DLBC, COAD, CHOL, CESC, BRCA, and BLCA, whereas a significantly positive connection in LGG, SARC, and THYM (**Figure 9A**). These results suggested that HIC1 expression may be correlated with immunotherapeutic responses in these human cancer types.

### Correlation between HIC1 expression with immunotherapeutic efficacy

The potential of HIC1 in predicting the immunotherapeutic efficacy of anti-PD-1/PD-L1 treatment for cancer patients was further investigated. A total of 3 cohorts, including GSE78220, GSE67501, and IMvigor210, were included in our study to compare the differential HIC1 expression between immunotherapy-responsive and immunotherapy-nonresponsive patients. The results showed that HIC1 expression was significantly higher in patients with nonresponses to immunotherapy in IMvigor210 (**Figure 9C**) and GSE78220 cohorts (**Figure 9D**), while there was no static significance in the GSE67501 cohort (**Figure 9E**). These results indicated that HIC1 could effectively predict the immunotherapy responses for individual cancer patient and might be a novel therapeutic target to overcome immunotherapy resistance.

### Drug sensitivity analysis of HIC1

**Figure 10:**
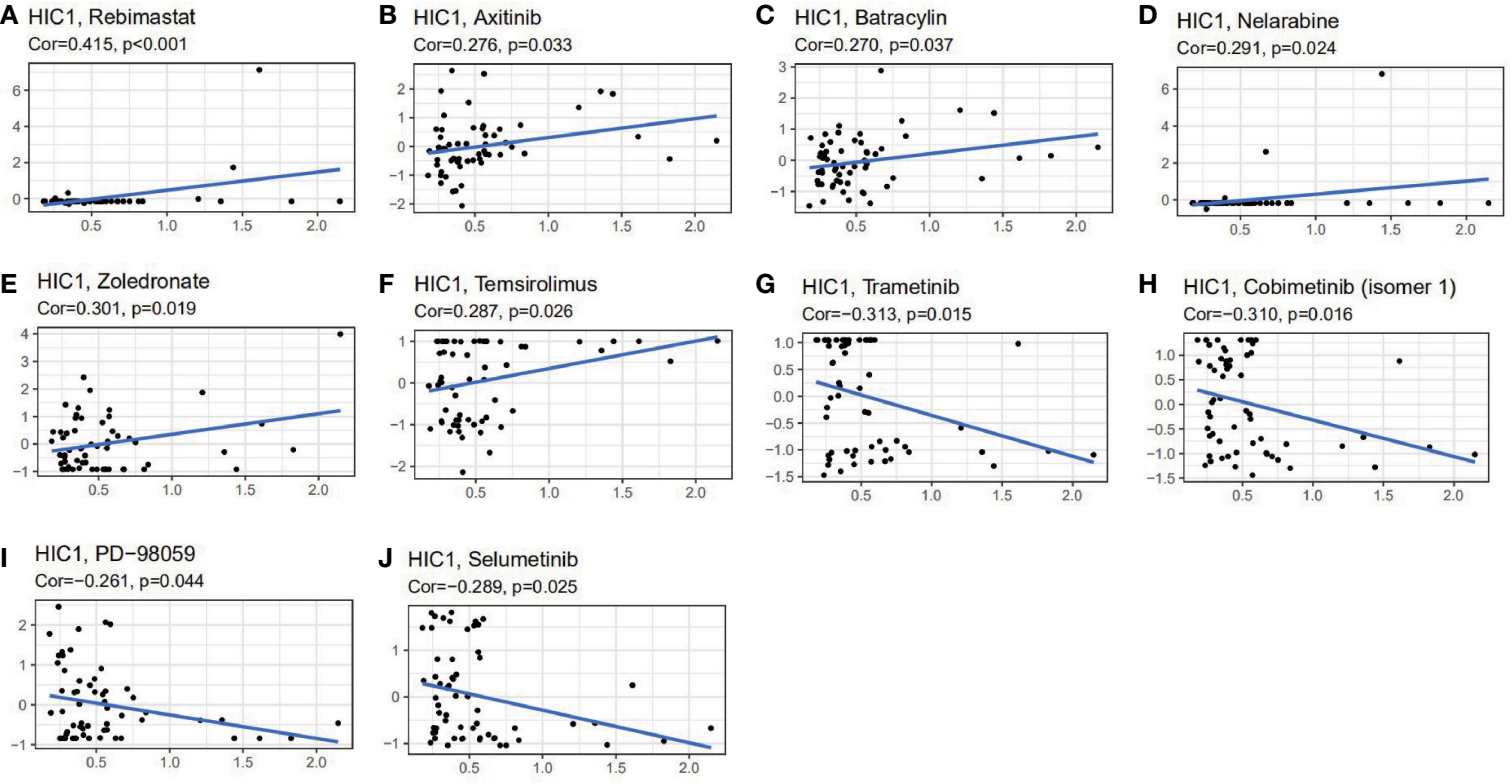
The correlation between HIC1 expression and drug sensitivity. The HIC1 was linked to the sensitivity of **(A)** Rebimastat, **(B)** Axitinib, **(C)** Batracylin, **(D)** Nelarabine, **(E)** Zoledronate, **(F)** Temsirolimus, **(G)** Trametinib, **(H)** Cobimetinib, **(I)** PD-98059, and **(J)** Selumetinib.

We further explored the potential relationship between HIC1 expression and drug sensitivity by utilizing the CellMiner database. We found that HIC1 expression was positively correlated with the sensitivity to several agents, including rebimastat, zoledronate, nelarabine, axitinib, temsirolimus, and batracylin (**Figures 10A–F**), while was negatively correlated with the sensitivity to trametinib, cobimetinib, selumetinib, and PD−98059 (**Figures 10G–J**). Notably, the results indicated that HIC1 might be significantly correlated with the sensitivity to several small molecule inhibitors that have been applied in cancer treatment, such as MEK inhibitors trametinib and PD−98059.

### IHC validation of HIC1

**Figure 11:**
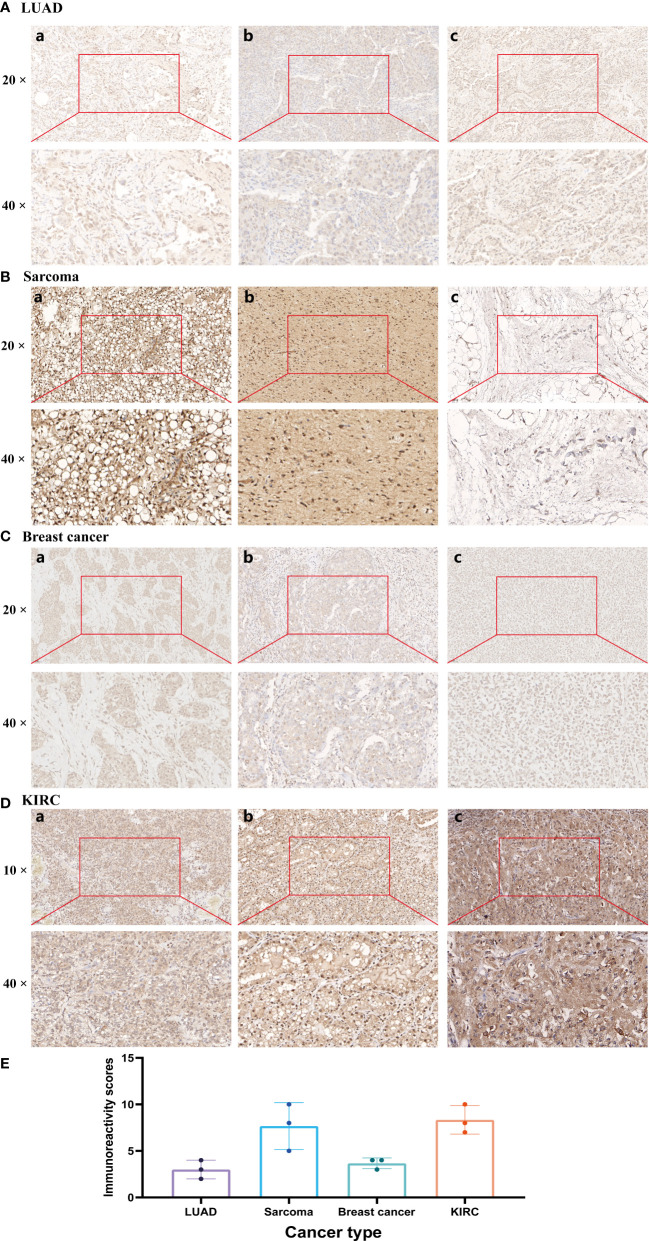
Immunohistochemistry validation of HIC1 in different cancers by clinical samples. **(A)** LUAD, **(B)** SARC, **(C)** Breast cancer, **(D)** KIRC, and **(E)** Immunoreactivity score.

The expression of HIC1 was further verified by IHC across 4 different types of cancer by our cohorts, including LUAD, SARC, breast cancer, and KIRC. As shown in **Figure 11**, HIC1 was detected in all of the examined tumor tissue samples. A strongly positive expression of HIC1 was observed in SARC and KIRC, while low expression of HIC1 was detected in patients with LUAD and breast cancer. The immunoreactivity score of each cancer type was presented in **Figure 11E**. These findings further were generally consistent with previous bioinformatics analysis. In addition, we also obtained IHC results from the HPA database and compared the results with HIC1 protein level in UALCAN. The IHC staining of HIC1 was mainly weakly or negatively expressed in tumor tissue from breast cancer, LUSC, LUAD, and COAD while was relatively higher in their corresponding normal tissues (**Figure S2**).

## Discussion

HIC1 is frequently hypermethylated which lead to the inactivation of HIC1 in the development of tumor. As a direct target gene of P53, HIC1 is associated with the regulation of cell-cycle regulation, thus contributing to tumorigenesis (22). A HIC1-SIRT1-p53 circular loop has been well illustrated. In the circular loop, HIC1 inhibits the transcription of SIRT1 which deacetylates and suppresses the expression of p53, thus resulting in the inactivation of HIC1 in turn (23). Nowadays, the role of HIC1 in several cancers, such as colorectal cancer (24), epithelial ovarian cancer (25) and medulloblastoma (26), have been investigated. Recent work has found that HIC1 can regulate ferroptosis during cancer progression (14, 15, 27). Wang et al. have constructed and validated a novel prognostic signature including 3 ferroptosis-related genes: HIC1, LPCAT3, and DUOX1. *In vitro* experiments revealed that inhibition of HIC1 can promote chemosensitivity and anti-PD1 therapy efficacy through inducing ferroptosis in ovarian cancer cells (15). Notably, ferroptosis has been confirmed to play critical roles in cancer immunotherapy, and ferroptosis activation may be a potential strategy to promote the immunotherapy efficacy (28). For instance, inhibition of APOC1 can increase the M1/M2 macrophage ratio through regulating ferroptosis and improve the anti-PD-1 immunotherapy efficacy for hepatocellular carcinoma (HCC) (29). Wang et al. have found that immunotherapy-activated CD8 + T cells can enhance ferroptosis-specific lipid peroxidation in tumor cells, and that increased ferroptosis further results in the increasing anti-tumor efficacy of immunotherapy (30). However, the roles of HIC1 are inconsistent and controversial among several cancers, there are no pan-cancer analysis of HIC1 and the associations of HIC1 with tumor immune microenvironment and the immunotherapeutic efficacy are still largely unknown. Therefore, we perform a pan-cancer analysis to thoroughly explore the clinical significance of HIC1 as well as its critical roles in tumor immune microenvironment and immunotherapy.

To begin with, we investigated the expression levels and clinical significance of HIC1 in different cancers. The results showed that compared with adjacent normal samples in TCGA, HIC1 expression was significantly decreased in tumor samples in BLCA, BRCA, CESC, COAD, KICH, KIRP, LUAD, LUSC, THCA and UCEC, while was strongly increased in CHOL, HNSC, and KIRC. By comparing HIC1 expression between TCGA tumor samples and GTEx normal samples, upregulation of HIC1 was also detected in GBM, LAML, PAAD, and STAD, and downregulation of HIC1 was also found in ACC, ESCA, LGG, LIHC, OV, PRAD, SKCM, and UCS. These inconsistent findings may be attributed to the application of different algorithms, sample sizes and sources in distinct databases, and the insufficient number of normal samples in TCGA may also result in insignificant findings in some cancer types. There was also disparity between HIC1 expression in TCGA tumor tissues and CCLE cancer cell lines, which may be attributed to the fact that there are multiple cell lines in a specific cancer type in CCLE database, and gene expression level is often significantly different among these cell lines *in vitro* experiment. Also, gene expression in cancer patients may change during cancer progression and after receive different treatment options, which may also explain this phenomenon. IHC results in our clinical samples further confirmed the low protein level of HIC1 in breast cancer and LUAD, while the relatively high HIC1 protein expression in KIRC and SARC, which further validates HIC1 expression pattern in bioinformatics analysis. Previous studies have found that HIC1 was downregulated in bladder cancer, and HIC1 can inhibit bladder cancer progression through the YAP signaling pathway (31). HIC1 expression has been found to be silenced only in triple-negative breast cancer compared with other breast cancer molecular subtypes, and HIC1 slicing could facilitate triple-negative breast cancer progression by targeting lipocalin-2 (LCN2) (32). However, Brieger et al. indicated that chromosome 17p13.3 where HIC1 is located on is a region usually lost in HNSC (33) and Eggers et al. showed that HIC1 hypermethylated and inactivated in KIRC (34). These results conflicted with our findings, possibly because of the different sources of samples and heterogeneity, which should be further validated in more large cohorts. With regards to the clinicopathological significance of HIC1, our results found that HIC1 was expressed higher in patients with later clinical stages in BLCA, ESCA, and STAD, indicating that HIC1 may function as a biomarker for predicting disease progression for cancer patients. Zhang et al. have reported that HIC1 expression was negatively related to the clinical stage in patients with liver cancer (14). As determined by pan-cancer prognostic analyses, high HIC1 expression was significantly correlated with improved OS, DFS, DSS, and PFS in ACC, OS, DSS, and PFS in UVM, DSS, and PFS in KIRP, OS in MESO, and TGCT. In contrast, UCEC patients with high HIC1 expression were significantly associated with worse OS DFS, DSS, and PFS. There were also significant associations between CHOL patients with high HIC1 expression and worse PFS, BLCA patients with worse DFS, and PCPG patients, and worse DSS. Combining these results, our studies suggest patients with high HIC1 expression had a better prognosis in ACC, UVM, KIRP, MESO, and TGCT, while had a worse prognosis in UCEC, BLCA, CHOL, and PCPG. Previous studies indicated that overexpression of HIC1 can act as a poor prognostic biomarker for KIRC, while a biomarker for better prognosis in pancreatic cancer (9, 34). It has been found that HIC1 is an important contributor to the development and functions of several immune cells, such as T cells and macrophage. Therefore, the associations of HIC1 with cancer prognosis may attribute to its ability to mediate the body’s immune response. Overall, these findings indicate that HIC1 may serve different functions in different cancer types, and is a critical prognostic biomarker in several cancers, and monitoring HIC1 expression may help predict the prognosis of cancer patients, which is mutually corroborated by previous studies.

Cancer is usually resulted from genetic alterations and cancer genomes included 4-5 mutations on average (35). Genetic changes play an important role in regulating cancer development and immune tolerance. For instance, mutant PD-L1 with structural variations can contribute to aberrant PD-L1 expression and immunosuppression. The amplifications of JAK2/PD-L1/PD-L2 (9p24.1) can induce constitutive overexpression of PD-L1 and a significant response to immune checkpoint inhibitors (36). In our study, we found that the major types of genetic alteration of HIC1 were deep deletion, amplification, and miss mutation. The genetic alternation frequency of HIC1 was higher than 2.5% in CHOL, which was matched with previous studies (37). It has been found that several CpG-islands (HIC1, OPCML, SFRP1, PTEN, and DcR1) presented a frequency of hypermethylation >28% of CHOL (37). In prostate carcinoma, a high frequency of alterations in the promoter methylation status of HIC1, SFRP2, and DAPK1 was detected in patients with prostate carcinomas of high Gleason Score (GS) (38). Currently, the functions of HIC1 genetic alternations in cancer immunological activity are still largely known and warranted further investigation.

To further explore the biological functions and downstream signaling pathways of HIC1 in different cancer types, we conducted KEGG and GO analysis. Our results suggested that HIC1 plays an important role in the chemokine signaling pathway in several cancers. It has been reported that deletion of HIC1 can contribute to premalignant transformation in the early stage of tumor formation. Moreover, the HIC1-deleted breast cancer cells can secret CXCL14 to its cognate receptor GPR85 on mammary fibroblasts in the microenvironment, and activate fibroblasts through the ERK1/2, Akt, and neddylation signaling pathways, whereas the activated fibroblasts can facilitate breast cancer progression through inducing epithelial-mesenchymal transition (EMT) by the CCL17/CCR4 axis (39). Besides, the results indicated that HIC1 is associated with T cell-related pathways, including the T cell receptor signaling pathway and T cell activation. Previous studies have reported that HIC1 suppresses the function of human induced regulatory T cells (iTreg) by interacting with the transcription factors (TFs) required for the development of Th1/2/17 cells (40). In addition, HIC1 can promote the differentiation of tissue-resident memory T cells (T_RM_ cells) (41). These findings are consistent with our results and confirmed the critical roles of HIC1 in mediating T cell functions. Our results also showed that HIC1 is related to cytokine-cytokine receptor interaction, calcium signaling pathway, MAPK signaling pathway as well as regulating the immune system.

The tumor immune microenvironment has been regarded as an integral part of cancer, which forms a complex tumor microenvironment that supports the growth and metastatic dissemination of cancer cells (42). Importantly, novel targets within the tumor immune microenvironment can help direct and improve the actions of cancer immunotherapies which can reshape the tumor immune microenvironment and restores the capability of immune cells to kill tumor cells. To further reveal the role of HIC1 in the tumor immune microenvironment, we first analyzed the relationship between HIC1 expression and immune and stromal scores by the ESTIMATE algorithm, which presented a positive correlation in multiple cancers, such as BLCA, CHOL, and COAD. Furthermore, we explored the relationship between HIC1 expression levels and the abundance of infiltrating immune cells in the specific cancer type. Our results showed that HIC1 expression was mainly associated with T cells, B cells, macrophages, and mast cells in the tumor immune microenvironment in multiple cancers. Previous studies have indicated the correlation between HIC1 and T cells, including iTreg and T_RM_ cells (40, 41). HIC1 has been found to be upregulated early during the differentiation of human iTreg cells, and HIC1 deficiency can contribute to a significant loss of suppression by iTreg cells with a concomitant upregulation of effector T cell associated genes (40). Besides, HIC1 has been reported to regulate the differentiation of B lymphocytes by inhibiting the transcription of class II transactivator (CIITA) (43). To date, little research has been conducted to investigate the role of HIC1 in mediating immune cells in tumor immune microenvironment, which may be a novel direction for exploring the biological functions in oncology.

TMB is defined as the total number of mutations present in a tumor specimen and reflects cancer mutation quantity (44). High TMB is clinically related to better response for immune checkpoint inhibitors (ICI) and has been acknowledged as a predictive biomarker (45). MSI is also a predictive biomarker for the responses of cancer patients to ICI (46). Narayan G et al. have suggested that the expression level of HIC1 is positively correlated with the frequency of MSI-H in cervical cancer (47). Our results indicated that the expression level of HIC1 is associated with TMB in 20 cancer types and MSI in 5 cancer types, such as STAD, READ, SKCM, and DLBC, suggesting the promising potential of HIC1 as a biomarker for predicting the efficacy of cancer immunotherapy. Furthermore, we identified the role of HIC1 in the immunotherapeutic efficacy in 3 cohorts. Our results illustrated that in patients with metastatic urothelial cancer and melanoma, there was a higher expression level of HIC1 in patients with no-response to PD-1/PD-L1 inhibitors, suggesting that HIC1 may serve as a promising biomarker for predicting the immunotherapy efficacy in melanoma and urothelial cancer. Several studies have explored the clinical significance and functions of HIC1 in metastatic urothelial cancer and melanoma. For instance, it has been reported that HIC1 prohibited the progression of uveal melanoma by activating lncRNA-numb, providing a potential therapeutic target for uveal melanoma (48). In KIRC, patients with lymph node metastases presented a low methylation level of HIC1 compared to patients without lymph node metastases, and hypermethylation of HIC1 can act as a poor prognostic biomarker for renal cell carcinoma (34). In addition, hypermethylation of the HIC1 exacerbated prostate cancer metastasis by inducing epithelial-mesenchymal transition (EMT) mediated by Slug and CXCR4, which contributed to the poor prognosis of prostate cancer patients (39). However, the predictive value of HIC1 for immunotherapeutic efficacy in these cancers has not been illustrated, which should be further investigated in future studies.

Therefore, HIC1 has the potential to act as a biomarker associated with cancer immunotherapies and predict immunotherapy responses in cancer patients. Dynamic monitoring of HIC1 expression may be a valuable approach to effectively evaluate the immunotherapeutic responses of cancer patients, thus helping choose the most suitable therapy strategy for individual cancer patients. Moreover, we also explore the relationship between HIC1 expression and the anti-cancer drug sensitivity of cancer patients. Koul S et al. have reported that the promoter hypermethylation of HIC1 was involved in the resistance of Male germ cell tumor (GCT) to cisplatin (49). Our findings showed that HIC1 is closely related to the sensitivity of multiple anti-cancer drugs, especially small molecule inhibitors, including MEK inhibitors trametinib and PD−98059, indicating HIC1 plays a critical role in predicting the sensitivity of anti-cancer drugs. Among these drugs whose sensitivity is related to HIC1 expression, zoledronate, and trametinib have been reported to be associated with ferroptosis. In osteosarcoma, zoledronic acid can induce ferroptosis by decreasing ubiquinone and upregulating the expression of HMOX1 or cytochrome P450 oxidoreductase (POR) (50, 51). Besides, zoledronic acid also induced ferroptosis in osteoclasts by suppressing ubiquitination and degradation of p53 through FBXO9 (52). In addition, the combination of the MEK inhibitor trametinib and the autophagy inhibitor hydroxychloroquine (HCQ) could inhibit proliferative activity in Lkb1-deficient Kras-driven lung tumors by inducing ferroptosis (53). Future studies should focus on the roles of HIC1 in mediating cancer drug resistance through the regulation of ferroptosis.

Though we have comprehensively conducted numerous analyses to illustrate and validate the roles of HIC1 in pan-cancer, there are still some limitations in our research. Firstly, although we have validated the expression pattern in our clinical samples, the associations of HIC1 with immunotherapeutic efficacy and anti-cancer drug sensitivity have not been validated in our own cohorts. Secondly, the specific mechanisms by which HIC1 regulates the tumor immune microenvironment remain largely unclear and have not been illustrated in experiments. Therefore, future studies are required to investigate the biological functions of HIC1 in tumor immune microenvironment.

## Conclusion

In summary, this comprehensive pan-cancer analysis of HIC1 reveals the expression pattern and role of the ferroptosis-related gene HIC1 in different cancer types. Our findings suggested that HIC1 may serve as a prognostic biomarker, and is related to immune infiltration, immunotherapeutic efficacy, and anti-cancer drug sensitivity in various cancers, thereby providing a theoretical basis for more precise cancer treatment in the future. Further research is needed to verify the specific mechanisms involved.

## Data availability statement

The original contributions presented in the study are included in the article/**Supplementary Material**. Further inquiries can be directed to the corresponding authors.

## Ethics statement

The studies involving human participants were reviewed and approved by the Ethics Committee of Second Xiangya Hospital. The patients/participants provided their written informed consent to participate in this study.

## Author contributions

YLW and ZJL: writing–original draft, data curation, and formal analysis. ZYT and ZHL: immunohistochemistry analysis. YQJ and ZXT: writing–original draft. YTX, and ZTZ: formal analysis. JY and TL: writing–review and editing, and visualization. TL and ZHL: writing–review and editing, supervision, and funding acquisition. All authors contributed to the article and approved the submitted version.

## Supplementary material

Supplementary Figure 1 A positive correlation between HIC1 expression and stromal scores in SARC, SKCM, STAD, TGCT, UCEC, OV, LIHC, LUAD, KIRP, HNSC, CESC, and BRCA.

Supplementary Figure 2 Comparison of HIC1 gene expression between normal and tumor tissues and immunohistochemistry images in normal and tumor tissues. **(A)** LUSC, **(B)** Breast cancer, **(C)** LUAD, and **(D)** COAD.
