# Supplementary figures and images for: Ferroptosis-related gene HIC1 in the prediction of the prognosis and immunotherapeutic efficacy with immunological activity
Source: Front Immunol. 2023 Jun 14;14:1182030. doi: 10.3389/fimmu.2023.1182030 (PMC10300279; doi:10.3389/fimmu.2023.1182030)

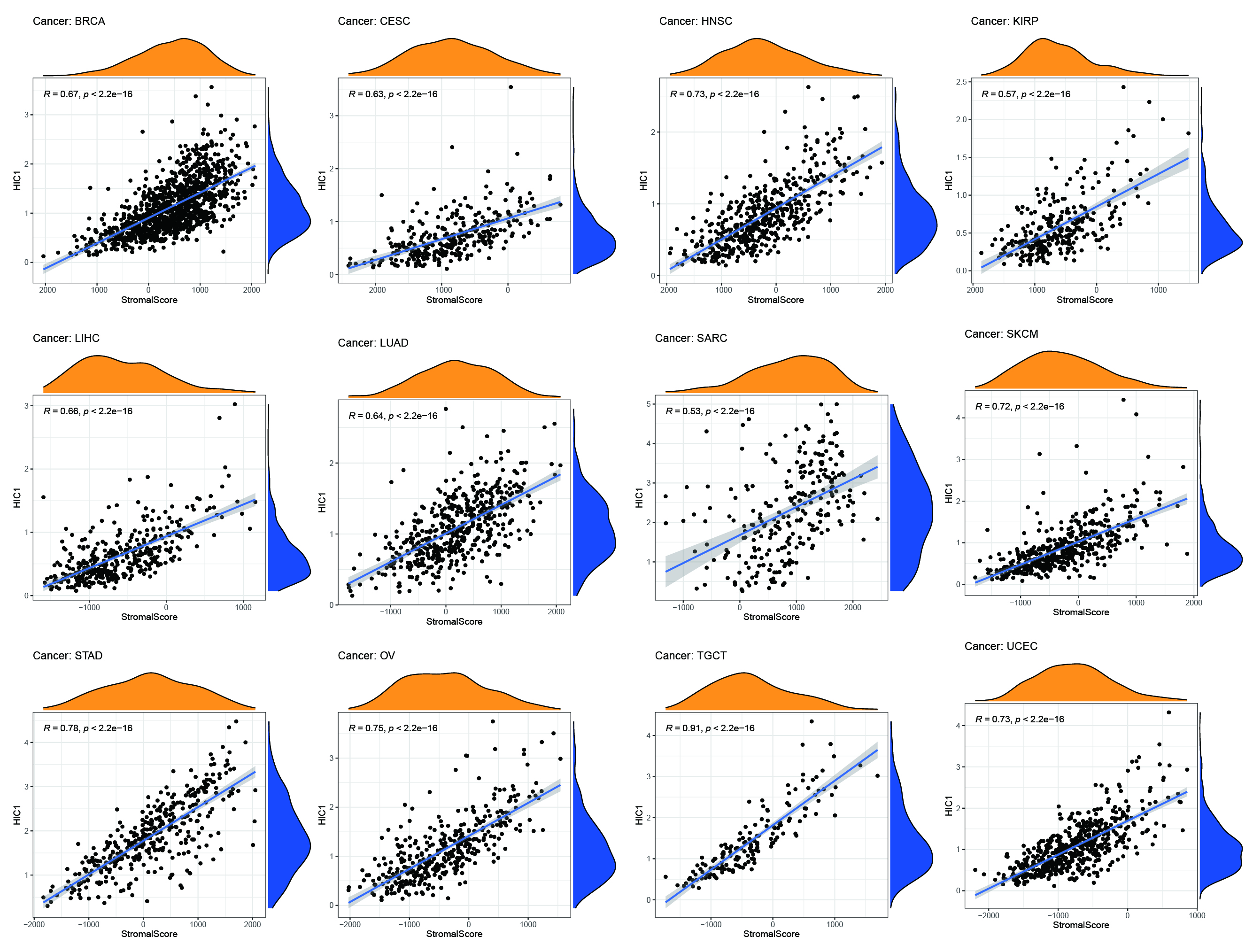

Supplement: Supplementary Figure 1 — A positive correlation between HIC1 expression and stromal scores in SARC, SKCM, STAD, TGCT, UCEC, OV, LIHC, LUAD, KIRP, HNSC, CESC, and BRCA. [file Image_1.jpeg]

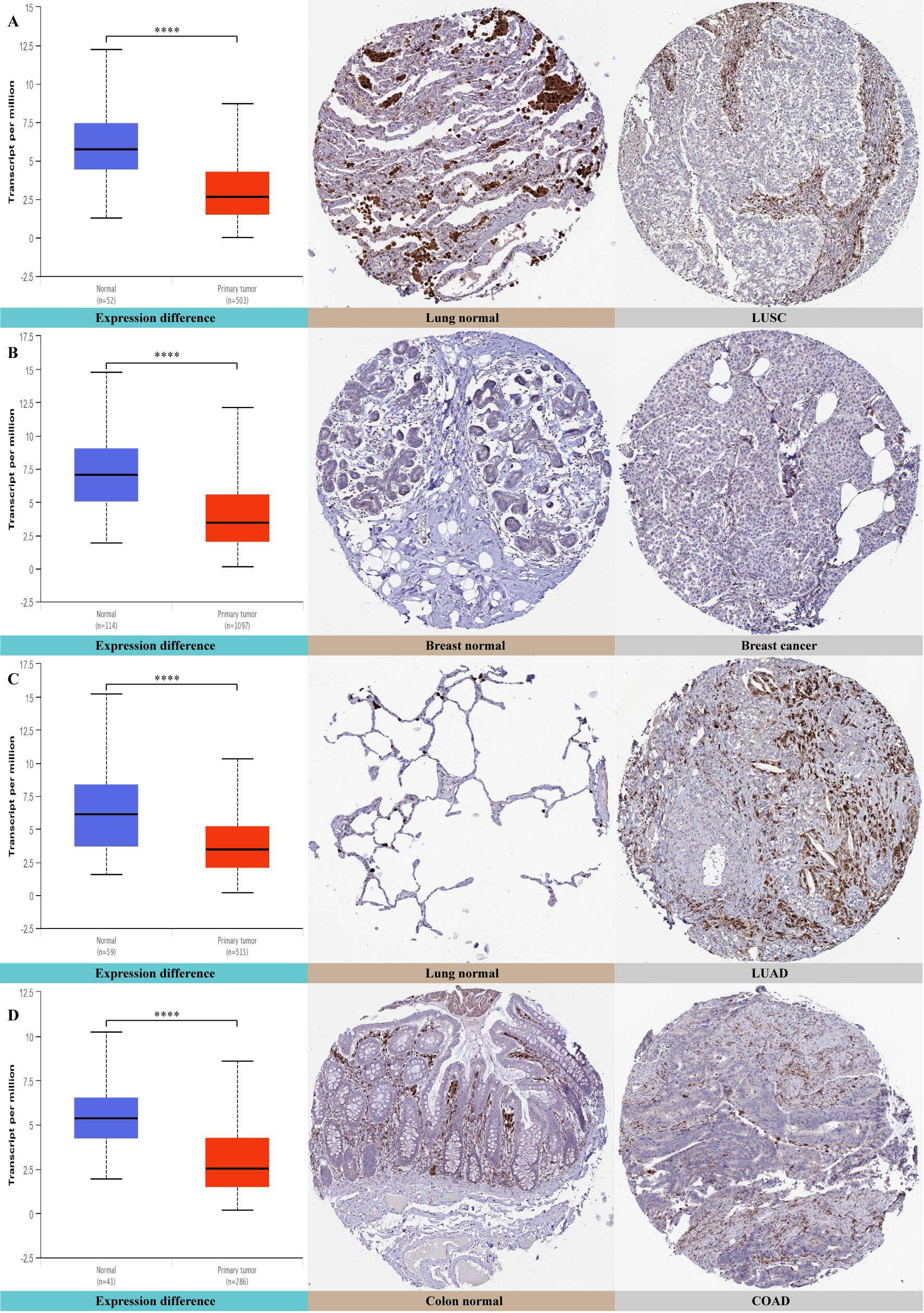

Supplement: Supplementary Figure 2 — Comparison of HIC1 gene expression between normal and tumor tissues and immunohistochemistry images in normal and tumor tissues. (A) LUSC, (B) Breast cancer, (C) LUAD, and (D) COAD. [file Image_2.jpeg]
