# Supplementary material for: Ferroptosis-related gene HIC1 in the prediction of the prognosis and immunotherapeutic efficacy with immunological activity
Source: Front Immunol. 2023 Jun 14;14:1182030. doi: 10.3389/fimmu.2023.1182030 (PMC10300279; doi:10.3389/fimmu.2023.1182030)
Supplement: Supplementary file 5 [file Table_2.docx]

**The links for raw data obtained from publicly available datasets in this article as follow:**

1. TCGA-pan-cancer project: https://xena.ucsc.edu/

2.CCLE database: https://portals.broadinstitute.org/ccle/about

3. http://www.sangerbox.com/tool

4. TISIDB database: http://cis.hku.hk/TISIDB/index.php

5. MSigDB database: https://www.gsea-msigdb.org/gsea/downloads.jsp

6. https://tcga.xenahubs.net

7. GEO dataset: https://www.ncbi.nlm. nih.gov/geo/

8. CallMiner database: https://discover.nci.nih.gov/cellminer/home.do

9. GTEx database: <https://www.gtexportal.org/>

10. cBio Cancer Genomics Portal: http://cbioportal.org
